# ID 33 - Características de Avaliações Econômicas Submetidas para Incorporação de Medicamentos no Sistema Único de Saúde: Um Estudo Metaepidemiológico

**DOI:** 10.5327/2237-9622.2025.v34s1.33

**Published:** 2025-11-25

**Authors:** Gilson Pires Dorneles, Cintia Pereira de Araujo, Bruna Marmett, Ana Paula Blankenheim, Marina Guahnon, Suena Parahiba, Cinara Stein, Maicon Falavigna

**Keywords:** avaliação econômica em saúde, reembolso, Conitec

## Abstract

**Introdução:**

Alocar recursos financeiros de forma eficiente representa um desafio significativo para os sistemas de saúde em todo o mundo. Nesse contexto, as avaliações econômicas em saúde (AES) são fundamentais para o processo de tomada de decisão da Comissão Nacional de Incorporação de Tecnologias no Sistema Único de Saúde (Conitec). O objetivo deste trabalho foi caracterizar aspectos metodológicos e a qualidade do relato de AES de dossiês submetidos à Conitec para incorporação de medicamentos no SUS.

**Método:**

Buscas eletrônicas foram conduzidas no repositório da Conitec para a identificação de dossiês para incorporação de medicamentos para o SUS publicizados entre 2020 a junho de 2024. A seleção dos documentos e a extração de dados foram realizadas para obter aspectos como as características do caso-base (condição clínica, intervenção, comparadores, horizonte temporal, desfechos em saúde, desfechos de custos), tipo de AES e de modelo matemático adotados, e também aspectos sobre a obtenção de utilidade em saúde e análise de incertezas do modelo. A qualidade do relato das AES foram avaliadas pelo instrumento 2022 (CHEERS 2022). A sumarização dos dados ocorreu por meio de estatística descritiva para apresentação de frequência absoluta e percentual.

**Resultados:**

188 AES de 180 dossiês foram incluídos neste estudo, sendo 46% com decisões favoráveis para incorporação. As principais áreas clínicas foram oncologia (19%), doenças raras (15%) ou infecciosas (13%). Cerca de 38% dos dossiês adotaram a combinação de análises de custo-efetividade e custo-utilidade, no entanto, uma parcela importante adotou análise de custo-minimização (23%). Dados sobre a data de extração dos custos ou ajustes dos custos pela inflação foram relatados por 60% e 28% dos dossiês, respectivamente. A maior parte das AES utilizaram o modelo de Markov (35%) ou análise de sobrevida particionada (16%) para modelagem econômica, enquanto análises de incertezas determinísticas (79%) e probabilísticas (88%) foram usualmente adotadas. Enquanto o plano de análise de custo-efetividade (91%) foi amplamente relatado, apenas metade dos dossiês relataram a curva de aceitabilidade de custo-efetividade (50%). Anos de vida ajustado pela qualidade foi o principal desfecho em saúde utilizado (95%) nas análises de custo-efetividade, porém aspectos como desutilidade para eventos adversos ao medicamento, desutilidade do cuidador, ajuste de dados de utilidade em saúde para a idade do caso-base ou para valores nacionais foram pouco abordados. Desfechos como sobrevida global e sobrevida livre de progressão foram também adotados nos dossiês que conduziram análises de custo-efetividade. A avaliação da qualidade do relato pela ferramenta CHEERS 2022 identificou que aspectos como plano de análise econômica, relato metodológico sobre custos das intervenções, caracterização da heterogeneidade e dos efeitos distributivos carecem de melhores descrições nos documentos.

**Conclusão:**

Este estudo destaca a necessidade de maior refinamento e padronização dos métodos de AES submetidos à Conitec. As diretrizes metodológicas vigentes para AES , que datam de 2014, estão atualmente em revisão. Esta atualização é crucial para integrar os avanços recentes e para melhor atender aos requisitos contemporâneos, especialmente frente ao novo limiar de disposição a pagar.

